# Development and validation of two composite aging measures using clinical biomarkers in the Chinese population

**DOI:** 10.1093/geroni/igaa057.3274

**Published:** 2020-12-16

**Authors:** Zuyun Liu

**Affiliations:** School of Public Health and the Second Affiliated Hospital, Zhejiang University School of Medicine, Hangzhou, China

## Abstract

Quantifying aging is crucial for addressing aging and related issues. This study aimed to: 1) develop two composite aging measures in the Chinese population using two recent advanced algorithms (the Klemera and Doubal method and Mahalanobis distance); and 2) validate the two measures by examining their associations with mortality and disease counts. Based on data from the China Nutrition and Health Survey 2009 wave (N=8,119, aged 20-79 years, 53.5% women), a nationwide prospective cohort study of the Chinese population, we developed Klemera and Doubal method-biological age (KDM-BA) and physiological dysregulation (PD, derived from Mahalanobis distance) using 12 routine clinical biomarkers. For the validation analysis, we used Cox proportional hazard regression models (for mortality) and linear, Poisson, and logistic regression models (for disease counts) to examine the associations. We replicated the validation analysis in the China Health and Retirement Longitudinal Study (CHARLS, N=9,304, aged 45-99 years, 53.4% women). We found that both aging measures were predictive of mortality after accounting for age and gender (KDM-BA, per one-year, HR=1.14, 95%CI=1.08, 1.19; PD, per one-SD, HR=1.50, 95%CI=1.33, 1.69). With few exceptions, these mortality predictions were robust across stratifications by age, gender, education, and health behaviors. The two aging measures were associated with disease counts both cross-sectionally and longitudinally. These results were generally replicable in CHARLS although four biomarkers were not available. In summary, we successfully developed and validated two composite aging measures‒‒KDM-BA and PD, which have great potentials for applications in early identifications and preventions of aging and aging related diseases in China.

